# Regional Deprivation and Non-Cancer Related Computed Tomography Use in Pediatric Patients in Germany: Cross-Sectional Analysis of Cohort Data

**DOI:** 10.1371/journal.pone.0153644

**Published:** 2016-04-18

**Authors:** Steffen Dreger, Lucian Krille, Werner Maier, Roman Pokora, Maria Blettner, Hajo Zeeb

**Affiliations:** 1 Leibniz Institute for Prevention Research and Epidemiology - BIPS, Bremen, Germany; 2 Institute for Medical Biostatistics, Epidemiology and Informatics, University Medical Center Mainz, Mainz, Germany; 3 International Agency for Research on Cancer, Lyon, France; 4 Helmholtz Zentrum München, German Research Center for Environmental Health (GmbH), Institute of Health Economics and Health Care Management, Neuherberg, Germany; 5 Health Sciences Bremen, University of Bremen, Bremen, Germany; Institute for Health & the Environment, UNITED STATES

## Abstract

**Background:**

Conflicting findings were observed in recent studies assessing the association between patients’ area-level socio-economic status and the received number of computed tomography (CT) examinations in children. The aim was to investigate the association between area-level socio-economic status and variation in CT examination practice for pediatric patients in Germany.

**Methods:**

Data from Radiology Information Systems for children aged 0 to < 15 years without cancer who had at least one CT examination between 2001 and 2010 were extracted in 20 hospitals across Germany. The small-area German Index of Multiple Deprivation (GIMD) was used to assess regional deprivation. The GIMD scores were classified into least, medium and most deprived areas and linked with the patient’s last known postal code. A multinomial logistic regression model was used to assess the association between patients’ CT numbers and regional deprivation adjusting for age, sex, and location of residence (urban/rural).

**Results:**

A total of 37,810 pediatric patients received 59,571 CT scans during the study period. 27,287 (72%) children received only one CT, while n = 885 (2.3%) received six or more. Increasing numbers of CT examinations in non-cancer patients were significantly associated with higher regional deprivation, which increased, although CI overlap, for higher CT categories: ‘2–3 CT’ odds ratio (OR) = 1.45, 95%CI: 1.40–1.50; ‘4–5 CT’ OR = 1.48, 95%CI: 1.38–1.59; ‘6+CT’ OR = 1.54, 95%CI: 1.41–1.69. In addition, male sex, higher age categories, and specific body regions were positively associated with increased numbers of CT examinations.

**Conclusion:**

We observed a positive association between regional deprivation and CT numbers in non-cancer pediatric patients. Limitations of the ecological approach and the lack of differentiation of CT details have to be acknowledged. More information on CT indications is necessary for a full assessment of this finding. In addition, further work on ways to assess socio-economic status more accurately may be required.

## Introduction

Ionizing radiation is associated with cancer, with risks increasing with higher doses and at younger ages at exposure [1]. In modern-day medicine ionizing radiation is increasingly used in diagnostic and therapeutic radiological procedures to diagnose and treat various diseases and injuries in patients of all ages including children. Next to conventional x-ray examinations, computed tomography (CT) imaging has been established as an essential diagnostic tool. It provides quick and detailed series of x-ray images of patients, and is particularly useful in emergency situations when rapid decision-making is required but is also used for many general indications including cancer diagnostics. CT scans, however, use substantially higher doses of ionizing radiation compared to conventional x-ray examinations, while diagnostic approaches such as ultrasound or magnetic resonance imaging (MRI) provide alternative diagnostic approaches not using ionizing radiation [2]. The use of these procedures has been steadily increasing worldwide for the past decades [3, 4]. In Germany, CT examination rates increased from 0.08 to 0.12 examinations per individual on average annually between 1996 and 2010, while numbers of conventional x-ray examinations declined at the same time [5]. These comparatively higher radiation doses are assumed to potentially increase children’s risk for cancer, as children are known to be more radiation sensitive and have a longer lifespan after first exposure to potentially develop malignancies [1].

Recent studies on childhood cancer and exposure to medical ionizing radiation from CT examinations suggest the possibility of elevated cancer risks [6–10]. Elevated risks for leukemia and brain cancer were observed in the UK [6], and Mathews and colleagues observed higher incidence rate ratios for all cancers combined after CT exposure than for non-exposed individuals in Australia [7]. Another study from Taiwan found an elevated risk of brain cancer in children who were exposed to CT examinations of the head. Risks of leukemia and all cancers combined were also increased [8]. The most recent studies from France and Germany specifically addressed issues from the previously reported studies relating to predisposing medical conditions, the potential for confounding by indication and reverse causation, and found lower, but also elevated cancer risks in exposed children [9, 10].

Associations between individual socio-economic status or regional deprivation and health outcomes are well-documented in many countries including Germany. Elevated rates of cancer, chronic diseases and injuries both in adults and in children have been found in more deprived areas [11–15]. These medical circumstances often require extensive diagnostic procedures including CT examinations. Some recent studies have considered the relation between CT scans and socio-economic status using area level deprivation indices as a proxy measure [7, 16, 17] but these results remain conflicting. A UK study found socio-economic variations in CT examination numbers of children and adolescents in Northern England, with individuals from deprived areas receiving higher numbers of CT scans [17]. Similarly, a recent Dutch study found a weak but positive association between socio-economic status of the area of residence and CT numbers, which was not statistically significant [16]: the lower the socio-economic status, the higher the number of examined individuals. In contrast, data of an Australian study indicated a weak negative trend in CT numbers, with children from least deprived areas receiving more CT examinations [7].

To better understand the relation of regional deprivation and CT use among children within the German healthcare system we used the German Index of Multiple Deprivation (GIMD) as a small area measure for socio-economic status and data from a retrospective hospital-based cohort study conducted in Germany [10]. The GIMD was recently adapted to the German context as a small area-based deprivation measure at the municipal and district level [13, 15].

## Methods

We used data from a large German cohort study which was set up to investigate childhood cancer risk after exposure to ionizing radiation from computed tomography [10]. In the participating 20 hospitals we extracted data from Radiology Information Systems for pediatric patients who had at least one CT examination in the age range between 0-<15 years (applying a 6-months latency period, the analyzed age range was 0–14.5 years). For this study we only considered data from 2001 to 2010 and only included CT examinations for non-cancer patients as multiple CT examinations may be necessary to arrive at a cancer diagnosis, which would lead to increased CT examination numbers in the process compared to other diseases or injuries. Cancer patients were identified through linking the study population with the German Childhood Cancer Registry, and included those children who had a prevalent cancer diagnosis before their first CT examination during the study period or who received a cancer diagnosis within two years after their last CT examination (i.e. 2-year latency period during follow-up). The collected data included patient’s date of birth, sex, postal code of location of residence at time of last know CT examination, as well as number, type and date of CT examination undergone.

We used the German Index of Multiple Deprivation (GIMD), which was established based on the method used in the UK to create the Indices of Multiple Deprivation [18]. The GIMD is based on official socio-demographic, socio-economic and environmental data. Most data are derived from the Federal Statistical Office of Germany and the statistical offices of the German federal states, and were mainly from the year 2006. The indicators based on this data were assigned to seven deprivation domains reflecting aspects of material and social deprivation (i.e. income, employment, education, municipal revenue, social capital, environment and security deprivation). These single domains, ranging from 0 (least deprived) to 100 (most deprived), were then weighted and combined to an overall deprivation index. More details on the methodology and the use of the GIMD have been published elsewhere [13–15]. For this study, we used the GIMD as area-level socio-economic status for each patient. The nation-wide GIMD was calculated for 9,620 municipalities in Germany. They constitute the lowest level of administrative division in Germany but vary greatly in population size, ranging from less than 100 inhabitants in small rural areas to cities with more than one million inhabitants. We classified the GIMD scores into terciles indicating least, medium and most deprived areas based on their frequency distribution across all German municipalities. We assigned the respective GIMD category to each patient by linking the residential postal code at time of last known CT examination to the municipal level via the corresponding official municipal key. We assigned the median GIMD category to each patient as postal codes do not always match one unique municipal key in Germany and may in some instances belong to three or more municipalities. Based on the patients’ postal codes, we further classified the location of their residence into urban and rural areas according to official settlement classification types (Federal Institute for Research on Building, Urban Affairs and Spatial Development (BBSR), http://www.bbsr.bund.de/BBSR/EN/Home) to assess associations with individual CT numbers.

CT examination numbers were counted during the study period and classified into five categories (1, 2–3, 4–5, 6–10 and 10+ CT examinations). To measure the effect of regional deprivation on patients’ CT examination numbers we employed a multinomial logistic regression model. As dependent variable we used four CT frequency categories (1, 2–3, 4–5 and 6+ examinations) because the highest category (10+ CT scans) in the initial classification had rather small numbers and was therefore combined with the second highest. A single CT examination event was set as reference category. We additionally adjusted for sex, age at time of first CT examination and location of residence (urban/rural). The contribution of each patient’s CT examinations in the model was weighted by their individual observation period during the study period because individuals with short observation periods (e.g. entering the cohort at age 13) will have by definition lower odds to have multiple CT examinations. The individual observation period was defined as the time between the date of first examination and the age of 14.5 years or the end of the study period (31-12-2010), whichever came first, and was used as a weight in the regression model. Furthermore, we used six categories of body regions to distinguish different types of examination: head and neck, chest, abdomen and pelvis, extremities, multiple regions, and other/unclassifiable. Stratified analysis by type of examination was additionally conducted to assess differences of CT numbers and deprivation categories adjusting for age and sex. Individuals with missing data (sex, address, type of examination) were excluded from analysis. Data management and statistical analyses were done using SAS 9.3 (SAS Institute, Cary, NC, USA).

### Ethics statement

This study was given ethical clearance by the ethical review committee of the medical chamber of Rhineland Palatinate and the data protection officer of the Mainz University Medical Center. Individual-level patient consent was not required as data were provided anonymized. Where required, additional approvals were obtained from the ethics committee/institutional review boards of the following participating hospitals: University Medical Center-UKSH Luebeck, Klinikum Oldenburg, Hannover Medical School, University Medical Center Goettingen, University Medical Center Mannheim, Dr von Haunerschen Kinderspital of the Ludwig-Maximilians-University Munich.

## Results

### Data management

A total of 59,657 CT examinations were recorded in 37,871 non-cancer pediatric patients younger than 15 years in participating hospitals between 2001 and 2010 in Germany. Details on sex were missing for 46 patients with 69 examinations, 15 patients with 17 examinations had a missing deprivation status due to invalid postal code information. We conducted a complete case analysis which included 37,810 pediatric patients with 59,571 CT examinations.

### Main results

#### Descriptive analyses

The mean age at time of first CT examination was 7.2 years and the majority of children were males (58.7%). The deprivation scores were similar in the study population (median: 18.0; interquartile range: 14.1–26.7) and for all municipalities in Germany (median: 18.9; interquartile range: 13.5–26.4), with a marginal overrepresentation of patients from medium deprived areas. Overall, 35,081 CT examinations were conducted in 22,194 male patients and 24,490 CT scans in 15,616 female patients (Table 1). N = 27,287 (72%) patients received only one CT examination, while n = 885 (2.3%) patients received six or more CT scans. The mean number of CT examinations was 1.58 (standard deviation: 1.48, range: 1–47). Most procedures were done in the oldest age group (10-<15 years) (40.8%) and only about 1% of CT examinations were conducted in children aged below 12 months at time of first CT examination. Two-thirds of all CT examinations were recorded in children living in medium and most deprived areas in Germany, and 71% of the study population lived in urban areas (Table 1).

**Table 1:** Study population characteristics of patients under 15 years of age who received CT examinations in Germany, 2001–2010.

	Patients		Examinations	
	(n = 37,810)		(n = 59,571)	
	n	%	n	%
**Number of CT examinations**				
*mean*: *1*.*6*, *SD ±1*.*5*				
1	27,287	72.17	27,287	45.81
2–3	8,176	21.62	18,340	30.79
4–5	1,462	3.87	6,298	10.57
6–10	712	1.88	5,116	8.59
>10	173	0.46	2,530	4.25
**Sex**				
Female	15,616	41.30	24,490	41.11
Male	22,194	58.70	35,081	59.89
**Age at time of first CT scan (in yrs.)**				
*mean*: *7*.*2*, *SD ±4*.*6*				
< 1	472	1.25	589	0.99
1–4	13,389	35.41	20,962	35.19
5–9	8,542	22.59	13,687	22.98
10 –< 15	15,407	40.75	24,333	40.85
**GIMD**				
*median*: 18.0*; IQR*: *14*.*1–26*.*7*				
Least deprived	12,549	33.19	19,228	32.28
Medium deprived	13,200	34.91	20,144	33.81
Most deprived	12,061	31.90	20,199	33.91
**Residential location**				
Urban	26,961	71.31	42,639	71.58
Rural	10,849	28.69	16,932	28.42

SD = standard deviation, IQR = interquartile range

CT examinations of the head and neck region accounted for the majority (69.2%) of all procedures during the study period, followed by chest examinations (12.4%; Table 2). Overall, there was a statistically significant association between the number of CT examinations for each type of examination and deprivation status (Table 2; p <0.05). A positive association was observed in particular for examinations of the ‘head and neck’, ‘chest’ and ‘multiple’ regions, with numbers of CT examinations increasing with higher deprivation status. These three examination types accounted for 86.9% of all examinations recorded during the study period. In contrast, scans of the abdomen and pelvis (5.8% of all examinations), extremities (4.6%) and other body/unclassifiable regions (4.4%) were negatively associated with deprivation status.

**Table 2:** Number of CT examinations by GIMD and type of examination in patients under 15 years of age in Germany, 2001–2010.

	GIMD				
Type of examination	Least	Medium	Most	Total	*(%)*
Head & Neck	12,951	13,679	13,856*	40,486	*(69*.*2)*
Chest	2,333	2,420	2,497*	7,250	*(12*.*4)*
Abdomen & Pelvis	1,153	1,137	1,115*	3,405	*(5*.*8)*
Extremities	932	957	794*	2,683	*(4*.*6)*
Multiple regions	883	1,085	1,145*	3,113	*(5*.*3)*
Other/unclassifiable	945	844	760	2,549	*(4*.*4)*
**Total**†	19,197	20,122	20,167	58,486	
*(%)*	*(32*.*8)*	*(34*.*4)*	*(34*.*5)*		

^†^ 85 scans had missing information on the type of examination

*p < 0.05 for association between mean number of CT examinations by type of scan and deprivation category (REF = least deprived) adjusted for age and sex.

Note: patients with more than one examined body part will appear in multiple categories.

#### Multinomial logistic regression analysis

The odds of children with two or more CT scans (reference category: single CT examination) was higher in the most deprived areas compared to least deprived areas, and increased with higher CT frequency categories [‘2–3 CT examinations’ odds ratio (OR) = 1.45, 95% confidence interval (95% CI): 1.40–1.50; ‘4–5 CT examinations’ OR = 1.48, 95% CI: 1.38–1.59; ‘6+ CT examinations’ OR = 1.54, 95% CI: 1.41–1.69], but these differences were not statistically significant between frequency categories (Table 3). Lower numbers of CT examinations were observed in children from medium deprived areas compared to those from least deprived areas in the lowest CT frequency categories (Table 3). Furthermore, male patients had higher numbers of CT examinations compared to females. Living in urban areas was significantly positively associated with increased odds to receive 2–3 examinations, whereas higher CT frequency categories were negatively associated. Higher age at time of first examination showed a positive association with CT examination numbers in the two highest CT frequency categories, in particular in the 4–5 CT scan group. In contrast, a negative association was observed in the lowest CT category for older children compared to children 12 months of age at time of first CT examination (Table 3).

**Table 3:** Adjusted multinomial logistic regression results: Odds ratio (OR) and 95% confidence intervals (95% CI) of CT numbers for three frequency categories (with 1 CT examination event as reference category, n = 27,287).

	2–3 CT examinations		4–5 CT examinations		6+ CT examinations	
	(n = 8,176)		(n = 1,462)		(n = 885)	
	vs.		vs.		vs.	
	1 CT examination		1 CT examination		1 CT examination	
	OR	95% CI	OR	95% CI	OR	95% CI
**GIMD**						
Least deprived	REF		REF		REF	
Medium deprived	0.91	(0.88–0.94)	0.92	(0.85–0.99)	1.34	(1.23–1.47)
Most deprived	1.45	(1.40–1.50)	1.48	(1.38–1.59)	1.54	(1.41–1.69)
**Sex**						
Female	REF		REF		REF	
Male	1.02	(1.00–1.05)	1.13	(1.07–1.19)	1.09	(1.02–1.17)
**Age at time of first CT (in yrs.)**						
< 1	REF		REF		REF	
1–4	1.01	(0.92–1.11)	4.19	(2.83–6.21)	1.79	(1.30–2.47)
5–9	0.94	(0.85–1.03)	4.28	(2.89–6.35)	1.73	(1.25–2.40)
10- < 15	0.85	(0.77–0.93)	3.57	(2.41–5.30)	1.31	(0.94–1.81)
**Residential location**						
Rural	REF		REF		REF	
Urban	1.10	(1.06–1.13)	0.98	(0.92–1.05)	0.82	(0.76–0.89)

## Discussion

The results of this study suggest that increasing numbers of CT in children aged below 15 years without cancer who underwent > = 1 CT were positively associated with regional deprivation in Germany, showing increased frequencies for higher examination numbers. In addition, male sex, higher age categories, and specific body regions (head and neck, chest, and multiple regions) were positively associated with increased numbers of CT examinations.

This is the first study using data from a large German cohort study to investigate associations between the number of CT examinations in non-cancer pediatric patients and socio-economic status using a small area deprivation index. As part of a nationwide epidemiological study involving major hospitals in different parts of Germany, we were able to consider detailed examination data for all included pediatric patients for the period 2001 to 2010. Additionally, we were able to exclude all cancer cases for this study which had been identified through linking the study population with the highly complete German Childhood Cancer Registry. As a consequence, we were able to assess CT use focusing on non-malignant examination indications such as trauma, injuries, chronic diseases or childhood disabilities because cancer diagnostics often use repeated CT imaging, which may lead to inflated CT examination numbers. Further, we used a multinomial modeling approach to investigate potential associations between CT examinations and deprivation status while adjusting for covariates including sex, age at time of first CT examination and location of residence (urban/rural). Alternative models did not fulfill underlying model assumptions. Finer CT frequency categorization were also tested and yielded essentially similar results.

Our study also had some limitations. The data we used were restricted to the study period 2001 to 2010 in order to ensure comparability with the German Index of Multiple Deprivation. As a consequence, we could not assess CT scans which occurred before this period, which may lead to some underestimation of CT examinations. A further limitation is that we were only able to consider CT scans conducted in the participating hospitals [19]. Previous studies conducted in Germany, however, showed that most CT examinations in children are conducted in hospitals [20]. Despite this evidence, it cannot be ruled out that participating hospitals referred patients to other specialists for follow-up examinations (from general to academic hospitals, academic to academic hospitals, or out of hospital practices). The follow-up could have included CT examinations, which would not have been part of our study, again leading to underestimation of CT.

As we did not have individual-level information, we used the GIMD on the municipal level as a measure for socio-economic status for each patient. The estimated effects should therefore be interpreted with caution as the aggregated data on the municipal level may not reflect individuals’ socio-economic status, but must be seen as an ecological association with CT use. Compared to the UK Index of Multiple Deprivation one advantage of its German adaptation with regard to health research is that the GIMD score does not include “health and disability” as an indicator which avoids bias in the correlation analysis. The municipalities included in our study varied markedly in population size. Patients living in medium deprived areas were marginally overrepresented compared to the whole of Germany, while CT examinations numbers were almost equally distributed across the deprivation terciles in our study. We assume that the distribution of the deprivation scores in the study population is not markedly skewed as the median and interquartile range of the deprivation scores of all municipalities in Germany was similar. Overall, one has to acknowledge the limited variation of deprivation categories in urban areas and in particular in larger cities which may have an effect on the direction of association. We do not suspect any oversampling of children from urban areas as their proportion in our subsample (71%) was consistent with the urbanization rate in Germany (74%, [21]).

Our observed patterns are not entirely comparable with previously published studies as we only considered non-cancer related CT examinations. However, sensitivity analysis with all children in the cohort including cancer cases confirmed the overall effect observed in the present study population. Our results are therefore compatible with those reported from the UK and also from the Netherlands where children living in deprived areas received more CT examinations [16, 17]. Mathews et al., however, showed a negative gradient in Australian children, with increasing numbers of exposed individuals in higher socio-economic status groups [7]. These differences may also be influenced by socio-economic differences in hospital emergency services use, which may increase the likelihood that individuals with lower SES obtain CT examinations in hospitals more frequently [22].

Young individuals, including children, living in deprived areas have been found to have higher injury rates [12, 23, 24]. For instance, a French study found higher rates of traffic accident-related injuries among children and young adults living in deprived areas in the Rhône Department [12]. A similar pattern was observed in a UK study, which found increased injury rates in particular among children from deprived urban areas [23]. As observed in our study, the increased numbers of CT examinations in children from urban areas could point towards specific living conditions in cities and towns. These circumstances may be linked to ‘riskier’ urban living environments compared to rural areas such as high traffic volumes or increased numbers of intersections in cities and towns. In contrast, the observed negative association with CT examination numbers for children from rural areas in the two highest CT categories may point towards higher proportions of individuals being examined with serious reasons for hospitalization (e.g. trauma) or may have higher morbidity such as chronic diseases or multiple disabilities which may require more CT examinations compared to those from urban areas.

The sex differences we observed are in line with findings of a recently published Dutch study on trends in pediatric trauma, which found higher male-to-female ratios for various injuries requiring hospital treatment [24]. For Germany, similar sex-related risk patterns were also observed in the latest report from the German Federal Office of Statistics [25]. Similar to the study from the UK [17], we found a positive association between older age groups and higher CT numbers in our study. In contrast, we observed a negative association in the lowest CT category. This trend may in fact depict the actual CT use pattern in the youngest patients. It can be assumed that particularly in the lowest age groups CT diagnostics may be superior to other imaging approaches such as ultrasound or MRI for quickly arriving at informative diagnostic results, compared to older children who are more capable to articulate pain and its location, and may be able to remain still for extended periods of time necessary for MRI examinations, thus potentially reducing the need for CT scans or opting for other diagnostic procedures. Overall, however, CT machines are increasingly and preferentially used in particular due to their rapid imaging capability and higher availability compared to MRI, which may not be operational 24/7 in most hospitals and additionally requires sedation of pediatric patients in order to ensure high image quality.

The frequency distribution of examined body regions in our study with head and neck CT examinations and scans of the chest comprising more than three-quarters of all examinations is consistent with studies from Australia, the UK, France, and the Netherlands [7, 9, 16, 17]. Injuries to the head are reported to be the most frequent reasons for hospitalization among children and adolescents in Germany [25], resulting from trauma such as automobile/bicycle accidents or falls in children as also observed in the Netherlands [24]. Further analysis stratified by residential location showed a statistically significant positive association with increasing deprivation status for all body regions for patients from urban areas (data not shown). In contrast, the direction of association changes for abdomen and pelvis, extremities, and multiple regions when both residential location types are considered, possibly indicating different living circumstances for patients from rural areas. However, caution is required in interpreting these results as variations in deprivation terciles for children from urban areas are limited due to the ecological nature and scale of the deprivation index.

In summary, this study found higher CT examination numbers in children without cancer living in more deprived areas in Germany. Further research into reasons for differences in the numbers of CT examinations is necessary including more detailed data on actual CT indications (e.g. specific diseases, trauma due to accidents). In addition, further work may be required to focus on assessing socio-economic status more accurately by incorporating individual-level information or by establishing and using deprivation status information for smaller scales (in particular for urban areas).
